# Nanotechnology-Enhanced Sunscreens: Balancing Efficacy, Safety, and Environmental Impact

**DOI:** 10.3390/pharmaceutics17081080

**Published:** 2025-08-21

**Authors:** Ruchi Khobragade, Anis Ahmad Chaudhary, Mohamed A. M. Ali, Mayur Kale, Neha Raut, Pratik Ghive, Hassan A. Rudayni, Krutika Nagpurkar, Milind Umekar, Rashmi Trivedi

**Affiliations:** 1 Department of Quality Assurance, Smt. Kishoritai Bhoyar College of Pharmacy, Kamptee 441002, Maharashtra, India; ruchirakeshk@gmail.com (R.K.);; 2 Department of Biology, College of Science, Imam Mohammad Ibn Saud Islamic University (IMSIU), Riyadh 11623, Saudi Arabiamamzaid@imamu.edu.sa (M.A.M.A.);; 3 Department of Pharmacology, Smt. Kishoritai Bhoyar College of Pharmacy, Kamptee 441002, Maharashtra, India; mayur.kale28@gmail.com; 4 Department of Pharmaceutics, Smt. Kishoritai Bhoyar College of Pharmacy, Kamptee 441002, Maharashtra, India

**Keywords:** nanotechnology in sunscreen, UV protection, eco-friendly ingredients, multifunctional formulations, skin barrier, skin physiology

## Abstract

Sunscreen protects skin from harmful Ultra Violet (UV) rays, preventing skin diseases like cancer and premature aging. This review explores the role of nanotechnology in enhancing sunscreen formulations by incorporating green and sustainable ingredients. Nanoparticles such as titanium dioxide and zinc oxide effectively reflect UV rays, improving protection while minimizing white residue, thereby enhancing aesthetics, stability, and efficacy. Recent advancements in formulation include lipid-based and polymer-based nanosystems that improve the delivery of active ingredients, offering multifunctional benefits. Additionally, modern sunscreens integrate anti-aging and antioxidant properties, reflecting the trend toward hybrid formulations with multiple skin benefits. The review also examines recent patents, highlighting innovations in nanotechnology-driven sunscreen formulations and delivery systems. Safety and regulatory concerns are critically analyzed, focusing on public perception of nanoparticles and their environmental impact. Issues such as manufacturing challenges and consumer hesitancy toward nano-scaled formulations due to safety considerations are also discussed. While nanotechnology presents significant potential in advancing sun protection, the review underscores the importance of balancing innovation with safety and sustainability. Ultimately, it serves as a guide for future research directions in nano-based sunscreens, advocating for responsible and informed development in the field.

## 1. Introduction

Sunscreen comprises agents that either absorb, reflect, or block ultraviolet (UV) radiation. They help prevent the damaging effects of exposure to such radiation on the skin, including premature aging, sagging, wrinkling, and excessive cell production associated with UV damage. The skin, in terms of weight and surface area, is the largest body organ, covering the body against sunlight. Overexposure to UV radiation, however, has deleterious effects, including impaired immune response, premature skin ageing, melanoma-causing redness, changes in skin pigmentation, sunburn, skin damage, precancerous growths, formation of both fine and deep wrinkles, and increased light sensitivity. The geographical location also plays a role in how badly the skin is damaged [1]. Examples of these include equatorial locations, for more likely serious skin damage as a result of the length of time of ultraviolet exposure, daily/seasonal variations in UV solar intensity, and host-dependent characteristics like age, skin pigmentation, immune state, and behaviour patterns. Over recent decades, the utilization of sunscreens has surged as awareness about the harmful impacts of extended sun exposure has grown [2]. The heightened use of sunscreen is associated with a deeper understanding of the dangers of prolonged sun exposure. Extensive research has confirmed that sunscreens offer substantial protection against skin cancer, notably melanoma and squamous cell carcinoma. Additionally, there is significant evidence indicating that sunscreens help prevent photoaging. Cosmetics are substances designed to be applied to the body surface or any part to alter the appearance [3]. Sunscreens fit this category as they are commonly used to protect the skin from UV radiation by being applied to the skin’s surface. Sunlight is a form of electromagnetic radiation that spans a continuous spectrum, which is commonly categorized into three primary wavelength ranges [4]. This review aims to systematically explore the role of nanotechnology in enhancing sunscreen efficacy, analyze its environmental and safety challenges, compare nanosystems based on delivery and performance, and identify gaps for future research and innovation.

Despite the growing interest in nanotechnology-based sunscreens, there remains a lack of comprehensive understanding regarding the balance between efficacy, safety, and environmental impact of these formulations. Most existing reviews focus on either the technological aspects or environmental safety in isolation, without providing an integrated analysis. Therefore, this review aims to bridge that gap by critically evaluating current nanocarrier systems used in sunscreens, comparing their performance in terms of UV protection, skin compatibility, and ecological safety. In doing so, it provides a holistic perspective on how nanotechnology can enhance sunscreen functionality while addressing regulatory, health, and sustainability concerns. This integrated approach helps inform researchers, formulators, and policymakers about the challenges and future directions in the field of nano-enabled sun protection [5].

**Figure 1:**
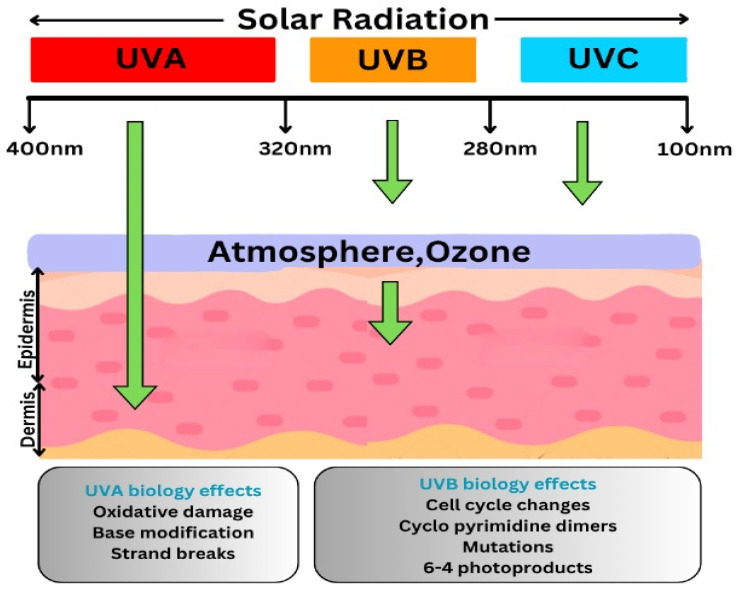
The Effects of UV Rays and Sunscreen UV Filters. It illustrates the effects of UV rays on the skin and the protective role of sunscreen UV filters. UV radiation may lead to skin deterioration, accelerate the aging process, and elevate the likelihood of developing skin cancer. Sunscreen UV filters help absorb, reflect, or scatter harmful UV rays, reducing their impact on the skin.

In the last decade, solar activity has notably increased, leading to a higher intensity of ultraviolet radiation reaching the Earth’s surface. Factors such as ozone layer thinning, climate change, and variations in solar cycles contribute to enhanced UV exposure, emphasizing the growing need for effective sun care strategies [6]. Ultraviolet A (UVA) radiation, which ranges from 320 to 400 nm, is further classified into UVA-I (340–400 nm) and UVA-II (320–340 nm). UVA-II is particularly harmful as it penetrates deeper into the skin and contributes significantly to photoaging and DNA damage. However, many conventional sunscreens struggle to effectively block the UVA-II range. Some advanced formulations that provide adequate UVA-II coverage carry a special UVA seal or “Broad Spectrum” label, especially in the European Union, indicating compliance with strict photoprotection standards [7] (Figure 1).

**Figure 2:**
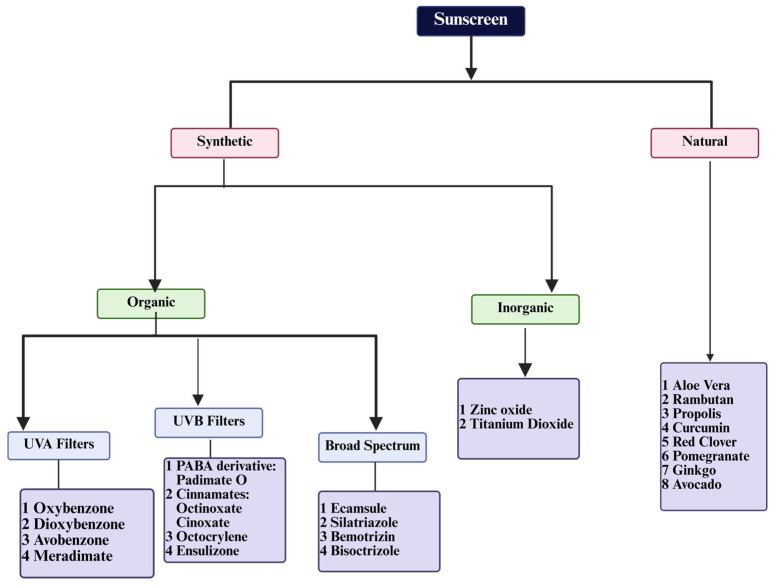
Classification of sunscreens based on their UV-filtering mechanisms. Sunscreens are classified into two types: organic (chemical) filters, which absorb ultraviolet radiation and transform it into heat, and inorganic (physical) filters, which deflect and disperse UV rays. Some formulations combine both types for broad-spectrum protection.

The types of Ultraviolet Rays are described in Table 1. An increase in cutaneous melanomas has led regulatory authorities to place greater expectations on the quality of sunscreen products as a result of worries about the quality of sunscreens [8]. This review examines the nature and classification of sunscreens, the science underlying their usage, their formulation and evaluation, and their historical context. Sunscreens are products designed to shield the skin from harm caused by UV radiation. They achieve this through active components that either deflect, absorb, or block UV rays [9]. Currently, sunscreens are widely recognized and used for photoprotection. Their UV defense capabilities are due to the presence of either organic or inorganic UV filters [10]. The sunscreens are classified in Figure 2.

The ozone layer completely filters out UVC rays. Both UVB and UVA radiation cause the melanin pigment to be produced, which gives rise, respectively, to a long-lasting and transient tan [12]. The effect of UV ray sunscreen filters is illustrated in Figure 1. As scientific knowledge and technologies have advanced, so too has the science of sunscreens, leading to improved formulations with respect to safety, efficacy, and aesthetic appeal [13].

## 2. UV Filters

### 2.1. Organic Filters

Organic UV filters contain an aromatic ring combined with functional groups that act as electron donors and acceptors. These molecular structures facilitate electron delocalization upon UV exposure, enabling effective absorption of ultraviolet radiation [14]. In child sunscreens, the most commonly utilized UV filters were triazine derivatives: bis-ethylhexyloxyphenol methoxyphenyl triazine (60.0%) and ethylhexyl triazone (52.0%), along with ethylhexyl salicylate (46.0%), which is a variant of salicylic acid. For adult sunscreens, the predominant filter was butyl methoxydibenzoylmethane (56.0%), a derivative of dibenzoylmethane, followed by the salicylic acid derivative ethylhexyl salicylate (54.7%) and triazine derivatives bis-ethylhexyloxyphenol methoxyphenyl triazine (54.7%) and ethylhexyl triazone (50.0%). Physical filters, in both their nano and non-nano forms, were favored in children’s sunscreens, with 50.0% (TiO_2_) and 22.0% (ZnO) usage, compared to adults: 21.3% (TiO_2_) and 6.7% (ZnO). Both adult and child cosmetic products typically contained four or five UV filters per formula; however, preparations for children often included just two UV filters. In conclusion, the UV filters that are prevalent in photoprotective products for both adults and children are: butyl methoxydibenzoylmethane, bis-ethylhexyloxyphenol methoxyphenyl triazine, ethylhexyl triazone, ethylhexyl salicylate, and diethylamino hydroxybenzoyl hexyl benzoate. Oxybenzone is a widely employed benzophenone that absorbs light rays [15]. Many UV filters are mixed to improve photostability and extend the protection level. Their structures are conducive to the absorption of UV radiation (UVR) and rejection of visible light (VL), followed by molecular shape alterations [16]. The absorbed energy is emitted as heat as the molecule changes from an excited state to the ground state [17].

### 2.2. Inorganic Filters

Zinc oxide (ZnO) and titanium dioxide (TiO_2_) are the two inorganic filters certified by the Food and Drug Administration (FDA), which are metal oxides known to absorb, reflect, or scatter EMR [18]. Distinct organic filters are non-toxic, hypoallergenic, and do not degrade through physical means. This, however, can put off the use of the products from the extended list of inorganic filters due to the fact that inorganic filters usually leave a whitish residue on the skin, which is a cause of concern, especially for individuals with Skin of Color (SOC) due to aesthetic reasons. Micronized versions of these filters enhance cosmetic appeal but may reduce their effectiveness in protecting against UVA and visible light (VL). In contrast, larger, more opaque pigments provide better defense against VL-induced photodermatoses, such as erythropoietic protoporphyria (EPP) [19].

## 3. Mechanism of Photoprotection

**Figure 3:**
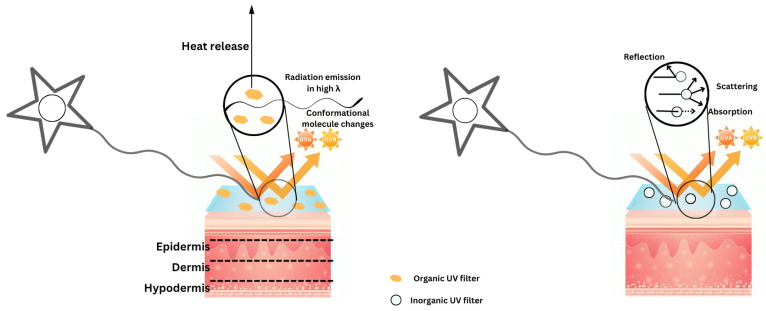
Mechanism of action of organic and inorganic sunscreens. Organic sunscreens absorb radiation of UV and convert it into heat, preventing skin damage. Inorganic (physical) sunscreens, Compounds like zinc oxide and titanium dioxide function as a protective shield, redirecting and dispersing UV radiation away from the skin.

Sunscreen actually works in two different ways. Below is a more detailed description of them [20]. UV radiation reflects off the skin’s surface and disperses (Figure 3). Mineral-based (inorganic) sunscreens operate in this manner. They form a protective barrier that prevents the skin from absorbing sunlight. The conversion of UV radiation into thermal energy upon absorption reduces the amount of damage and the depth at which the skin can be exposed to it (Figure 3). This is the primary way that organic sunscreens work [11].

In addition to conventional UV filters, SPF boosters have emerged as innovative ingredients that enhance the efficacy of sunscreens without increasing the concentration of active UV filters. SPF boosters function by improving the spreadability, film formation, and distribution of UV filters on the skin, leading to better coverage and reduced gaps in the protective layer. They do not absorb or reflect UV radiation themselves, but they optimize the performance of existing filters [21].

Examples of SPF boosters include:

- Butyloctyl salicylate—improves solubility and photostability of UV filters.
- Terephthalylidene dicamphor sulfonic acid (Mexoryl SX)—often acts synergistically with other filters to enhance UVA protection.
- Polyester-8—enhances UVB filter performance and boosts SPF while offering water resistance.
- Glyceryl stearate, hydroxyethyl acrylate/sodium acryloyldimethyl taurate copolymer, and silicone elastomers improve film uniformity and substantivity.
- Microfine zinc oxide or titanium dioxide, in combination with certain emollients or dispersing agents, improves skin adhesion and enhances broad-spectrum protection.

SPF boosters are particularly valuable for reducing the required amount of traditional UV filters, which can be irritating or environmentally harmful at higher concentrations. Moreover, by improving photostability, they help prevent degradation of active filters under prolonged sun exposure, increasing the durability and consistency of protection [22].

## 4. Eco-Friendly and Sustainable Ingredients in Sunscreen

Environmental concerns surrounding cosmetic ingredients have sparked an increasing demand for sustainable alternatives [23]. Some ingredients have been found to harm ecosystems, particularly aquatic life, leading to a global push for ecological goods, resulting in growing renewable, naturally sourced components, often labeled as biocosmetics, to align with consumers’ expectations of sustainability [24]. While individuals with higher education levels tend to be more conscious of environmental issues tied to cosmetics, many consumers are hesitant to pay a premium for sustainable options. The COVID-19 pandemic has further influenced consumer behavior, emphasized a healthier lifestyle, and created new opportunities in the cosmetic industry, particularly through circular economy practices [25]. The improper disposal of cosmetic products has raised environmental concerns. Substances like triclosan, zinc oxide, silver nanoparticles, squalene, and micro/nanoplastics tend to build up in aquatic habitats, impacting algae and disturbing ecosystems by modifying reactive oxygen species (ROS) levels, shifting biomass alignment that can harm pathogens at various trophic stages [26]. Sunscreens, while essential for preventing conditions like skin cancer and sunburn, also pose risks to aquatic ecosystems. UV filters can accumulate in organisms and contribute to pollution in water bodies such as rivers, lakes, groundwater, and seas [27]. Studies have shown that organic UV filters, like benzophenone-8 and 4-methylbenzylidene camphor, can be toxic to marine species, such as *Balanus amphitrite*. These pollutants are more concentrated in industrial and urban regions, though bioaccumulation can lead to long-term environmental issues even in remote areas. Further research is needed to understand the full scope of these impacts, as the environment’s complexity can significantly influence outcomes. The growing interest in natural alternatives for consumer-focused cosmetic formulations has prompted extensive research into natural sources for sunscreen applications [28]. Studies have indicated that incorporating natural ingredients into sunscreens can enhance their photoprotective effectiveness, primarily due to their antioxidant properties. These natural compounds help mitigate UV-induced skin inflammation, damage to the skin barrier, and signs of aging. Since UVA radiation triggers oxidative stress through reactive oxygen species (ROS), prolonged skin exposure can result in oxidative DNA damage [29]. Both topical and systemic antioxidants have been explored to counteract the effects of UV-induced oxidative stress. These antioxidants function by counteracting the damage induced by reactive oxygen species (ROS), helping to prevent or lessen tissue harm while also supporting tissue recovery after UV exposure. Certain naturally derived pure compounds, including lutein, flavonoids, pycnogenol, and lycopene, as well as plant extracts such as tea and fern extracts, are utilized. Research has shown that these substances provide protection against various types of UV-induced skin damage. In addition to terrestrial sources, marine biodiversity offers a largely untapped reservoir of natural UVR-screening compounds that hold potential for use in cosmeceutical formulations. These marine-derived substances present eco-friendly and safer alternatives to synthetic UV filters. Algae are particularly well-known for containing photoprotective compounds. Beyond algae, other marine organisms, including *Microorganisms*, *Artemia*, and *Plankton*, have also demonstrated photoprotective properties, expanding the scope of marine-based UV protection [30].

### 4.1. Emerging Trends in Sustainable Sunscreens

Recent advancements in nanotechnology and sustainability have catalysed a new generation of sunscreen formulations that go beyond basic UV protection. These innovations aim to address environmental, safety, and efficacy concerns by incorporating natural sources, biodegradable materials, and advanced targeting mechanisms [31]. Notable emerging trends include:

#### 4.1.1. Marine-Derived Nanomaterials

Marine ecosystems offer a vast and underutilized source of photoprotective compounds. Natural UV-absorbing substances such as mycosporine-like amino acids (MAAs), scytonemin, phlorotannins, and carotenoids derived from *Microalgae*, *Cyanobacteria*, and marine plants exhibit strong antioxidant and photoprotective properties. These marine-derived compounds are biodegradable and pose significantly lower environmental risks compared to synthetic UV filters. For example, MAAs from *Red algae* have shown potent UVB absorption and have been incorporated into liposomal and hydrogel systems for improved skin delivery [30].

#### 4.1.2. Biodegradable Nanocarriers

The incorporation of biodegradable nanocarriers, such as those made from polylactic acid (PLA), polycaprolactone (PCL), chitosan, and alginate, is gaining popularity due to their ability to degrade into non-toxic byproducts after application. These carriers not only reduce the risk of nanoparticle accumulation in marine environments but also enhance the controlled release of active ingredients. For instance, chitosan-based nanoparticles loaded with botanical UV filters like quercetin or resveratrol have demonstrated superior skin permeation and photostability [32].

#### 4.1.3. Blue-Light and Infrared Protection

While traditional sunscreens focus mainly on UV radiation, new research has emphasized the damaging effects of visible blue light (400–500 nm) and infrared radiation (700–1400 nm), both of which contribute to oxidative stress, pigmentation, and skin aging. Advanced formulations now include iron oxides, lutein, and niacinamide encapsulated in nanosystems to filter blue light and suppress ROS generation. These agents are often combined with antioxidant-rich nanoformulations to offer comprehensive photoprotection across the full solar spectrum [33].

#### 4.1.4. Green Nanotechnology Approaches

“Green synthesis” of nanoparticles using plant extracts, microorganisms, or biopolymers is a rapidly developing area. This approach eliminates toxic chemicals and reduces energy consumption during synthesis. For example, green-synthesized zinc oxide nanoparticles using Aloe vera or tea extract have shown comparable UV-blocking efficacy with improved biocompatibility. Additionally, lifecycle analysis of green-manufactured nanosunscreens suggests a lower carbon footprint and reduced ecotoxicity [34].

#### 4.1.5. Hybrid Natural-Synthetic Systems

Hybrid nanosystems combining natural photoprotective agents with FDA-approved filters are emerging as a strategy to enhance efficacy while minimizing potential side effects. These systems utilize biodegradable lipids or polymers to co-encapsulate ingredients like avobenzone with polyphenols or carotenoids, improving stability and reducing skin irritation.

#### 4.1.6. Edible and Ingestible Sunscreens

Although still in experimental phases, ingestible formulations containing nanoencapsulated astaxanthin, beta-carotene, or lycopene are being explored for systemic photoprotection. These ingredients, when delivered in lipid-based nanosystems like solid lipid nanoparticles (SLNs), offer a dual benefit: systemic antioxidant effects and UV resistance from within, reducing reliance on topical agents [35].

Table 2 provides an overview of the most commonly used natural cosmetic ingredients sourced from terrestrial plants, along with their particular INCI names & descriptions.

## 5. Nanosystems

In recent years, nanotechnology-based products have gained increasing interest within the cosmetics industry. The incorporation of nanoparticles (NPs) into cosmetic formulations offers several advantages: (i) improved stability and efficacy of the formulations, (ii) enhanced diffusion of active ingredients into the layer of skin, and (iii) functioning as UV filters. Cosmetic nanomaterials are classified into four primary categories: lipid-based nanostructures, polymer-based nanostructures, metal-based nanostructures, and other distinct nanotechnology systems [50].

### 5.1. Nanosystems Containing Lipid

Lipid-containing nanosystems proved highly effective as dermal carriers, offering advantages such as biocompatibility, stability, enhanced penetration, efficient delivery of active ingredients, and adaptability to various innovative dosage forms [51]. Their increased surface area facilitates better penetration of active compounds into skin tissues, making them a promising approach for targeted drug delivery to diverse therapeutic sites [52].

#### 5.1.1. Lipid Nanoparticles

##### Solid Lipid Nanoparticles and Nanostructured Lipid Carriers

A range of nanosystems facilitates precise drug delivery. In cosmetics, lipid nanoparticles gained considerable interest because of their well-established manufacturing methods, scalability, and desirable characteristics. Two generations of lipid nanosystems can be identified based on their lipophilic matrix structure. The first-generation, Solid Lipid Nanoparticles (SLNs), are mainly composed of solid lipids. SLNs provide a controlled-release property and minimize skin irritation by reducing the immediate release of active ingredients on the skin [53]. The lipids used in Solid Lipid Nanoparticles (SLNs) are solid at room temperature, which is advantageous for encapsulation and controlled release of active ingredients. SLN efficiency is affected by a variety of factors, including the production process and the physicochemical characteristics of the active ingredient, especially its lipophilicity [54]. Crystalline rearrangements in Solid Lipid Nanoparticles (SLNs) would take place during their storage, causing the formation of a more ordered and stable structure. This might lower matrix imperfections and lessen the room for the entrapment of active ingredients that might lead to their leakage or loss. To overcome the limitations of SLNs, another generation of lipid nanoparticles was developed as Nanostructured Lipid Carriers (NLCs) [55]. In such a way, NLCs combine solid and liquid lipids, generating a disordered matrix that will increase the incorporation of active ingredients and prevent leakage on storage, attributed to its amorphous structure. Both SLNs and NLCs can remain closely attached to the skin, enhancing their penetration through the stratum corneum. As occlusive agents, these alter the lipid structure of the stratum corneum, enhancing skin hydration, penetration of nano systems, and therapeutic efficacy. Besides, SLNs offer UV resistance, providing add-on photoprotective benefits [56].

#### 5.1.2. Vesicle-Based Systems

Vesicular structures containing encapsulated drugs provide an exciting means for enhancing dermatological therapies. These structures prove suitable for delivering different active ingredients throughout the different stratifications of skin in dermatological practices. Depending on lipid phase composition, preparation methods, and the nature of the drug, various vesicles with markedly varied physicochemical properties and therapeutic effects can be produced.

#### 5.1.3. Liposome Structures

##### Niosomes

Alternative systems, such as niosomes, were introduced to boost the therapeutic efficacy of liposomes. Niosomes are self-assembled nanovesicles that look like liposomes but have different compositions. Whereas liposomes are composed of lipids with phospholipid bilayers, niosomes are founded upon non-ionic surfactants to form bilayers. Surfactant molecules can change the structure of the stratum corneum lipid bilayer to increase skin permeability. Niosomes are usually prepared using cholesterol or polyethylene glycol (PEG) to enhance bilayer rigidity, stability, and mechanical strength, thus further improving performance. Niosomes contain a hydrophilic core and a hydrophobic bilayer; hence, they can encapsulate hydrophilic and hydrophobic ingredients. This unique ability makes niosomes suitable for active ingredient-delivering applications due to their versatility [57]. Niosomes have been utilized since the 1980s for encapsulating biomacromolecules like proteins, owing to their low toxicity, excellent compatibility with human tissues, and cost-efficiency, especially for chemotherapy applications [58]. Ethosomes, commonly found in various formulations, are primarily used in dermatological, beauty, and hair care cosmetic products. These specialized nanoparticles, ranging in size from 30 nm to microns, are designed to penetrate intact skin layers and efficiently deliver both hydrophilic and hydrophobic active ingredients. Ethosomes are made primarily of phospholipids, ethanol, and water, which contribute to their flexible structure. Upon hydration, this flexible structure allows ethosomes to get through the skin promptly, increasing the efficacy of their incorporated active ingredients [59]. Ethosomes are of three kinds: classical, binary, and transethosomes. Classical ethosomes represent an enhancement of liposome design by incorporating phospholipids and a high concentration of ethanol (45% *w*/*w*), resulting in improved entrapment capacity and enhanced transdermal delivery. Binary ethosomes differ by using an alcohol other than ethanol. Transethosomes are formulated with surfactant to enhance vesicle penetration, making them the most flexible vesicular systems for drug delivery [60].

Deep penetration and distribution are purportedly facilitated by the high ethanol concentration in these carriers, leading to fusion with skin lipid domains. Ethanol will help fluidize the lipid bilayer, while changes in lipid structure in the skin will increase the supplied active ingredients deeper in the skin. Ethosomes are important because they are easy to produce, highly effective in active ingredient delivery, and safer, thus offering a viable option in many pharmaceutical and cosmetic applications [61].

##### Transfersomes

Transfersomes are nanoparticles featuring a hydrophilic core encased in a lipophilic bilayer. Transfersomes penetrate the skin by utilizing the water compartment between the skin surface. An osmotic gradient then drives them deeper into the subcutaneous layers, enabling the delivery of active ingredients. Edge activators are materials like Tweens, sodium cholate, dipotassium glycyrrhizinate, or Spans that increase flexibility. These surfactants with single chains disrupt the lipid bilayer and increase the deformability of vesicles [62]. Transfersomes have various advantages: their relatively simple manufacturing process, flexible characteristics, and enhanced biocompatibility make them a valuable nanocarrier. These vesicles bring both the proposed low molecular weight molecules and the large ones, acting against them to provide protection from degradation and allow adequate release of active substances. The capacity to carry both hydrophobic and hydrophilic molecules enables the transport of these vesicles to a wide variety of substances with different solubility, adding to their versatility as drug delivery vehicles [63].

##### Cubosomes

Cubosomes are self-organized liquid crystal-like substances made up of certain lipids embedded in 3D continuous bilayers, like a honeycomb. This architecture allows for increased surface areas while maintaining its sustained release properties. The core of cubosomes contains two aqueous channels that facilitate the encapsulation of the active ingredients to ensure effective delivery [64]. Cubosomes possess unique characteristics that make them exceedingly useful, including their reduced viscosity as well as distinct structural properties that set the stage for the generation of specific-function formulations. Their ability to ensure sustained release of active ingredients, targeted delivery, and fantastic encapsulation efficiency. They are also noted for their ease of preparation and thermodynamic stability. Hence, cubosome engineering is taking shape very rapidly and could turn into a much-promising tool for pharmaceutical and cosmetic formulations soon [65].

#### 5.1.4. Nanoemulsions

Nanoemulsions are nanoscale systems consisting of droplets ranging from 25 nm to 550 nm. These are unbalanced and require the usage of both a surfactant and a cosurfactant, along with an energy input in thermodynamic form. To ensure the stability of the nanoemulsion, surfactants should be carefully selected to reduce interfacial tension, preventing physical instability issues such as sedimentation, flocculation, and coalescence. Nanoemulsions, which can be structured as biphasic oil-in-water (O/W), water-in-oil (W/O), or multiple phases (W/O/W or O/W/O), are capable of efficiently delivering both hydrophilic and lipophilic molecules [66]. Compared to microemulsions, smaller droplet size and the reduced amount of surfactant necessary have proved to be advantages of nanoemulsions that make them ideal for topical applications. The oily phase in nanoemulsions acts as an occlusive agent through which it preserves moisture in the skin and breaks the lipid bilayer of the stratum corneum, thus increasing the absorption of topically applied drugs. Low toxicity and viscous nature have led to an increased expansion of nanoemulsions in the cosmetic sector, with special interest in hair care preparations [67].

### 5.2. Polymer-Based Nanosystems

#### 5.2.1. Polymeric Nanoparticles

Nanoparticles are widely researched nano systems, generally ranging from 210 to 310 nm. Key factors influencing the delivery of active ingredients include. The appeal of polymeric nanoparticles lies in their ability to modify the physicochemical properties of active ingredients, thereby controlling their efficacy. These nanoparticles act as “containers for lipophilic drugs,”. Polymeric nanoparticles can be classified as nanocapsules or nanospheres, depending on the formulation components and preparation methods. This is done by encapsulating either hydrophilic or lipophilic active ingredients into a polymeric wall surrounding a core. The oily core might consist of solid, liquid lipids. NPs are made as colloidal suspensions that are processed into powders and gels to enhance their physical stability. Due to their unique morphology, these nanoparticles boast several advantages for cosmetic applications. Their small size enables much deeper penetration into the target tissues, and their surface characteristics allow for prolonged action in the body, which, in turn, favors targeting. This nanostructure also enables a controlled release of the active ingredients at the site of action with the least possible side effects [68]. Nanospheres are nanoparticles specifically designed to penetrate the deeper layers of the skin. Primarily consisting of a matrix, such systems have considerable promise for application in cosmetic purposes. These structures have relevant roles in the delivery of active components directly into the skin. Their unique design with interstitial spaces also contributes to their effective action. This allows better targeting and prolonged activity of the active components in the skin [69]. Nanoparticles made of polymers are highly promising for drug delivery, providing excellent stability. Within the cosmetic industry, they have entered the consumer market by being incorporated into skin care formulations [70]. Chitosan is the most widely used natural biopolymer in nanotechnology. It is obtained from the deacetylation of chitin; the latter being obtained mainly in the exoskeleton of crustaceans. It has its application in the development of polymeric nanoparticles for several purposes, spanning drug delivery and cosmetic formulations, formats carried out due to its biocompatibility, biodegradability, and capacity to form stable nanoparticles [71]. Because it can assist in promoting healing and has intrinsic antimicrobial activity, chitosan is of value for renewability, biocompatibility, and biodegradability. The antimicrobial effect depends on factors such as temperature, pH, deacetylation degree, and molecular weight. Due to the weak basic nature of chitosan, it interacts easily with carboxylic groups, forming salts. This quality gives it the potential of binding with hyaluronic acid, extensively used in the cosmetic industry for anti-wrinkle treatment, thereby offering significant chances for the creation of novel cosmetic products [72].

Lignin, a widely available and renewable polyphenolic polymer sourced from plant biomass, has great potential as a material in nanotechnology-based sunscreen products due to its natural ability to absorb ultraviolet (UV) light and its strong antioxidant characteristics. When transformed into lignin nanoparticles (LNPs), lignin enhances protection against a broad spectrum of UV radiation by effectively absorbing and scattering both UVA and UVB rays, which significantly boosts the sun protection factor (SPF) of formulations. Additionally, these nanoparticles enhance the stability of chemical UV filters like avobenzone and octyl methoxycinnamate by minimizing their degradation when exposed to UV light [73]. The antioxidant properties of lignin nanoparticles also assist in neutralizing free radicals generated by UV exposure, providing added protective benefits for skin health. The morphology and size of the particles play a crucial role in their ability to block UV rays, with research indicating that advanced shapes such as triangular lignin nanoparticles offer improved SPF enhancement and colloidal stability without causing whitening [74]. Beyond their functional benefits, lignin nanoparticles promote eco-friendly and sustainable sun care solutions, making them particularly suitable for dark-tinted SPF cosmetics because of their natural dark color, which avoids the whitening issues prevalent with inorganic filters [75]. Recent developments also highlight scalable green synthesis techniques and effective incorporation of lignin nanoparticles into sunscreen products that achieve high SPF levels while ensuring environmental safety and compatibility with the skin [73]. Taken together, these qualities position lignin nanoparticles as multifunctional, natural polymers that improve both the effectiveness and sustainability of contemporary sunscreen formulations.

#### 5.2.2. Nanofibers

To be sure, these are highly anticipated nanosystems in the cosmetic arena due to their unconventional properties. These fiber sizes can reach 500 nm in diameter, thereby offering a high surface area, a porous structure, and a favorable set of physical and mechanical properties that provide an ideal nanofiber for both hydrophobic and hydrophilic active cosmetic ingredients (ACIs) in controlled release. Their application addresses systemic absorption and reduces the required dosage of active ingredients in skin-care formulations. These fibers are made from natural polymers (collagen, silk, and chitosan) or synthetic polymers (e.g., PLGA, PVA, or PVP), and their efficacy largely depends on porosity, morphology, the type of polymer used, and size. The integration of natural and synthetic polymers in nanofiber formulations today capitalizes on the biological advantages of natural substances and the adaptable nature of synthetic polymers [76].

### 5.3. Metal-Based Nanosystems

Nanotechnology is a quickly developing field, and recently, inorganic metal and metal oxide nanoparticles have begun to appear within the cosmetic industry, especially in topical formulations. Such nanoparticles are ideal for novel delivery of active substances. However, they are not without their disadvantages; despite their advantages, NPs pose a risk from toxicological perspectives. Hence, ongoing research is currently assessing and remedying these unfortunate risks. These nanoparticles are increasingly found in various cosmetic products, including herbal formulations, sunscreens, hair care products, and makeup. Below are some of the most commonly used metal-based nanoparticles in cosmetic formulations [50].

#### 5.3.1. Silver Nanoparticles

Silver nanoparticles (AgNPs) are widely incorporated into consumer products due to their broad-spectrum antimicrobial and antifungal properties. Because of the increase in concern about antibiotic resistance, AgNPs offer a solution to this issue. The antimicrobial activity of AgNPs depends on cellular membrane alteration affecting permeability, which refers to their bactericidal ability. Once transported into the cell, the silver ions impair cellular respiration and promote the elevated generation of reactive oxygen species (ROS), responsible for the inhibition of bacterial growth. They also bind to the phosphorus and sulfur groups of DNA to cause unwinding and interference with both transcription and translation. Importantly, with the size of AgNPs, the antimicrobial activity was inversely proportional; that is, smaller-sized AgNPs with larger surface area seemed to be more effective. AgNPs find applications in cosmetic products like toothpaste, creams and lotions, soaps, deodorants, etc. [77].

#### 5.3.2. Gold Nanoparticles

Gold nanoparticles (AuNPs) have appeared exciting to the research community because of the various potential applications and even more because their size varies from 5 to 400 nm. In dermal delivery systems and biomedical engineering, AuNPs are considered valuable because of their unique chemical and physical properties, e.g., their capability to be modified in terms of shape, size, and crystallinity, ensuring a high drug load. This quality of penetrating through the skin, thus delivering active ingredients, makes AuNPs an interesting option for the cosmetic industry. It is also being studied with respect to properties like antioxidants, antimicrobial action, skin firming, elasticity strengthening, and delay in the aging process. They are now being incorporated into topical cosmetics [78].

#### 5.3.3. Titanium Oxide and Zinc Oxide Nanoparticles

TiO_2_ and ZnO nanoparticles are widely used in cosmetic products. TiO_2_ primarily reflects UVB radiation, while ZnO is effective against UVA radiation. The combined use of these two nanoparticles offers enhanced sun protection along with key benefits such as transparency, ease of application, and improved texture, all without skin irritation commonly caused by traditional chemical UV filters. The UV-blocking capability of these systems is particularly advantageous, as NPs remain on the outermost layer of the skin, the stratum corneum, providing effective protection from harmful sun exposure [79]. The antimicrobial action of ZnO is attributed to the generation of reactive oxygen species (ROS), which leads to the release of Zn^2+^ ions. These ions are cytotoxic to bacteria and disrupt their cell walls. When combined with titanium dioxide (TiO_2_), ZnO demonstrates a synergistic antimicrobial effect. However, the effectiveness of ZnO nanoparticles can be influenced by the other ingredients in topical formulations, as certain compounds like antioxidants or EDTA can interact with ZnO and reduce its antimicrobial activity [80].

#### 5.3.4. Silica Nanoparticles

Silica nanoparticles (SiO_2_ NPs) gained noteworthy attention from the cosmetic industry. These nanoparticles, with sizes ranging from 5 to 100 nm, are stable nanodispersions that can encapsulate both lipophilic and hydrophilic substances, making them effective for targeted delivery. Additional advantages include lower production costs and the flexibility of silane chemistry. Silica NPs also provide sustained release capabilities. Silica NPs occur in toothpaste, makeup, hair styling products, deodorants, and skincare products; these can act as emulsifiers, emollients, or water barriers. In addition, they enhance the spreadability of the sunscreen formulation, improve the sun protection ability, and reduce phototoxicity and degradation of sunscreen formulations [10].

### 5.4. Additional Nanosystems

#### 5.4.1. Dendrimers

Dendrimers are clearly structured spherical structures of extensive branches growing from a central core. This distinguishing morphology is crucial to their versatility. These are controlled to one side by their size and surface charge for inside routes. Their properties could be tuned by modifying the structure, permitting the production of dendrimers with special characteristics of interest. Moreover, the provided attachment of functional groups to the outermost branches could further lead to the tuning of such properties and contribute to their application potential. Additionally, their capacity to trap actives underscores their role in new formulations in the field of cosmetics. Thus, encapsulated in the core, it allows both hydrophilic and hydrophobic active cosmetic ingredients to dissolve in water without additional excipients’ intervention and organic solvents. The risk reduction for water degradation is dramatic here. Dendrimers facilitate skin absorption of active ingredients in that they interact with the lipid bilayers in the skin. The high cargo capacity further improves stability and dermal penetration of the ACIs. Therefore, dendrimers are featured widely in cosmetic applications ranging from skin to hair [81]. Dendrimers have an effective ability to entrap active cosmetic ingredients (ACIs) into their core, which is very advantageous for the cosmetic industry, providing a whole new range of formulations. The most hydrophilic as well as poorly soluble ones can be dissolved without allowing any additional excipients or organic solvents. Dendrimers boost the absorbance of active ingredients through skin modifications, whereby they interact with the lipid bilayers of the skin. Such high entrapment ability also means stability and much higher permeability of Credits over the epidermal layer. Thus, dendrimers are widely used in cosmetic formulations, encompassing the care of both skin and hair [82].

#### 5.4.2. Nanocrystals

Nanocrystals represent a class of newer nanoparticles with unique properties that make them particularly welcome additions in the field. These particles are general molecular-sized drugs trapped in dissolved colloidal state mixtures along with their stabilizers. They are made by milling bulk materials down to nano-sized ranges. Their presence seems to be similar to that of polymer nanoparticles-they have far greater loading capacities of drugs and thus can deliver drugs in more concentrated forms. Their size, along with enhanced solubility, ensures a comparatively higher rate of dissolution that gives rise to concentration gradients promoting faster ingress of active ingredients into the tortuous tissues. In addition, a larger surface area on their part ensures adhesion, which would ensure prolonged residency times at the target site [83].

#### 5.4.3. Fullerenes

Fullerene is a roughly spherical molecule made of 60 carbon atoms. Another type of fullerenes is fully commercially utilized because of one powerful quality-it is stable. Fullerene has the property of being an electron acceptor, giving it the ability to bind to free radicals, thus making it a potent antioxidant. Many studies have focused on its use in skin-whitening products and sunscreens. Fullerene may inhibit morphological changes in cells, prevent apoptosis, and inhibit melanogenesis. It also acts as a skin protectant by preventing keratinocyte differentiation, and its antioxidant action may promote hair growth, especially in cases due to oxidative stress. Furthermore, fullerene can penetrate the epidermis, making it an excellent carrier for active cosmetic ingredients (ACIs), as in acne treatments, where it reduces sebum production and displays antimicrobial activity against *Propionibacterium acnes* [84].

#### 5.4.4. Nanodiamonds

Nanodiamonds are nanoparticles containing a diamond core of nearly 5 nm, with functional groups attached to their surface. These nanoparticles have gained high market value mainly due to their stability resulting from the oxygen groups at the surface. Their unique structure, coupled with surface characteristics, makes these nanodiamonds a potential candidate for targeted delivery of molecules and drugs in cosmetics [85]. Many researchers opt for nanodiamonds because of their utility in modifying surface properties and interaction with biological systems to enhance the effectiveness of cosmetic treatments. Nanodiamonds will function well as effective nanosystems for the delivery of active cosmetic ingredients (ACIs) permeated through their bid to the skin tissues owing to their small particles [86].

#### 5.4.5. Cyclodextrins

Cyclodextrin is a cyclic oligosaccharide that consists of glucopyranose units linked together in some manner by α-(1,4) bonds. The complexation may change the physical and chemical properties of the encapsulated active ingredients and may improve the compounds’ stability, solubility, and bioavailability. Due to their amazing ability to prevent oxidation and its damaging effects on the skin, cyclodextrins are extensively used in cosmetic products. Cyclodextrins protect active cosmetic ingredients (ACIs) from degradation by forming inclusion complexes, thereby improving their stability. This is more important for volatile compounds in perfumes because it improves longevity and fragrance [87] Table 3.

## 6. Patents [88,89,90]

The recent patents of sunscreen are described in Table 4.

## 7. Safety and Regulatory Aspects of Sunscreen Formulation:

In 1943, para-aminobenzoic acid (PABA) was patented in Germany and France and became a widely used ingredient in sunscreens. Currently, sunscreens are regulated in many developed nations, with each country setting its own approved list of UV filters and determining the maximum permissible concentrations. Regulatory bodies set specific guidelines for sunscreen ingredients to ensure safety and effectiveness [89]. In India, sunscreen products are mainly controlled by the Central Drugs Standard Control Organization (CDSCO). Sunscreens are classified as cosmetics under the Drugs and Cosmetics Act, 1940, rather than as drugs, which means they face different regulatory requirements [90]. The CDSCO adheres to the guidelines that the Ministry of Health and Family Welfare set forth to guarantee the safety, quality, and effectiveness of products in India. It operates similarly to the FDA in the United States and is responsible for licensing, import authorizations, and post-market monitoring of drugs and cosmetics within the country. REGULATION (EC) No 1223/2009 OF THE EUROPEAN PARLIAMENT AND OF THE COUNCIL, dated 30 November 2009, pertains to the primary legislation regulating cosmetic products in the European Union (EU). This regulation establishes safety, labeling, and marketing standards for cosmetics available in the EU market, with the aim of safeguarding human health while harmonizing regulations across EU member nations. Additionally, this regulation outlines requirements such as restrictions on ingredients and product safety standards. Unlike drugs, which necessitate rigorous clinical trials and comprehensive safety data, cosmetics like sunscreens follow a less stringent approval process. This classification influences the standards that sunscreens must meet before entering the market, focusing more on safety and effectiveness without the extensive clinical trials required for pharmaceuticals. To market sunscreen products in India, manufacturers must secure an Import Registration Certificate from the CDSCO for imported products and a manufacturing license from the relevant State Licensing Authority (SLA) for domestic products [93]. Sunscreen formulations must include UV filters that are approved by regulatory standards, which are often based on international guidelines but may differ from those in regions like the USA or the EU [94]. A significant gap in the regulation is the absence of a maximum limit for SPF values, which can potentially lead to manufacturers making exaggerated claims. The lack of stringent testing protocols to validate these claims has been a point of concern in ensuring consumer safety and product efficacy. Labeling requirements are also specified under the Drugs and Cosmetics Rules, mandating that labels include essential information such as the product name, SPF value, usage instructions, and warnings about potential allergens or skin sensitivities. Recent regulatory updates have streamlined the registration process for cosmetics, allowing only 50 products per submission through the SUGAM portal to enhance efficiency while maintaining oversight. Despite these regulations, challenges remain in terms of standardization, particularly because UV filters are not included under Schedule S of the Drugs and Cosmetics Rules, resulting in a lack of prescribed quality standards [95]. Additionally, consumer awareness about sun protection is rising; however, many consumers may not fully understand product claims or ingredient safety. The growing interest in herbal and natural sunscreens offers an occasion as well as a challenge for regulation, since the assertion of the extra benefits of these products that emerge from a non-stringent scientific basis is rampant. The regulatory environment of sunscreens in India is changing, although continuous updates will be needed to protect consumers and also provide sun safety measures to the growing market’s expectations. The EU has categorized chemicals used in sunscreens (or UV filters) as cosmetic ingredients, thus governed by a positive list [96]. The list is regularly updated according to scientific research done under the aegis of the European Cosmetics Association (COLIPA), which works with the European Commission to keep pace with the most rigorous safety and efficacy standards. The positive list contains the UV filters permitted for use in cosmetic products and also the upper limits on the concentrations of each specific ingredient. This provides a framework under which the safety of sunscreens on the EU market could be ensured alongside transparency and consumer protection [97]. By classification, the regulation requires that sunscreen be classified as an over-the-counter (OTC) drug. For the acceptance of these drugs, the manufacturers must prove the effectiveness of their active ingredients, as well as their safety. It is the responsibility of the FDA to supervise and regulate these products, publishing monographs in the Federal Register. These monographs outline the approved active ingredients, concentration limits, and specific testing protocols that must be followed to market sunscreens. These guidelines have been periodically updated since their inception in 1978 to reflect new scientific findings [98]. The difference in approval reflects varying regulatory standards between the two countries, with the U.S. FDA requiring extensive data and testing for ingredient safety, while India’s regulations are still evolving and may have different standards for approving sunscreen chemicals [99]. In Canada, sunscreen products are categorized as either prescription drugs or natural health products depending on their ingredients. Health Canada recently introduced a draft revision of the “Sunburn Protectants Monograph”, now titled “Guidance Document Sunscreen Monograph”. This revised document is intended to supersede the 2006 Sunburn Protectants Monograph. The revision is designed to ensure sunscreen products meet rigorous standards for safety and effectiveness before being imported, advertised, or sold in Canada [100]. Canada has a broader list of approved sunscreen chemicals compared to India.

In Australia, due to the unusually high rates of skin cancer in that country, sunscreen plays an important part in daily sun protection. Many Australians regularly apply sunscreen over widely exposed areas of their bodies. The Therapeutic Goods Administration (TGA) controls sunscreens as therapeutic goods to ensure safety, efficacy, and quality. The list of approved sunscreen ingredients published by the TGA was last updated in 2006, providing a list of allowed sunscreen agents for use in sunscreens that are marketed as health products. This is supposed to ensure protection against harmful UV radiation. The Australian Regulatory Guidelines for Sunscreens (ARGS) provide very useful guidance for manufacturers and sponsors on regulatory requirements for sunscreens in Australia. They are meant to make certain that sunscreen products are assessed for safety, efficacy, and quality before they are made available to the market. The areas dealt with include marketing of active ingredients, labeling requirements, testing protocols, and sun protection claims. The ARGS also set out which sunscreens are considered therapeutic goods and what steps need to be followed in order to gain approval from the TGA. The guidelines ensure that the sunscreens in Australia offer adequate protection from UV radiation while following the approval standards [101]. In Australia, sunscreens are classified into three classes based on their SPF rating. Registrable sunscreens make therapeutic claims such as skin cancer prevention or a reduction in the risk of skin damage and therefore undergo a more thorough evaluation before receiving a stamp of approval. On the contrary, exempt sunscreens make no health-related claims and are usually slightly lower in SPF [102].

The TGA oversees the approval and regulation of sunscreen products in Australia. Sunscreens must contain only approved ingredients that have been assessed for safety and efficacy. Each product must undergo SPF testing to confirm its sun protection factor, which is then displayed on the label. Several UV filters are approved for use in Australian sunscreens but are not included in India’s list of approved ingredients. These include Benzophenone-1, -2, -4, Dioxybenzone, Cinoxate, Menthyl anthranilate, Tinosorb S, Zinc oxide, Salicylic acid salts (Potassium, sodium, triethanolamine), Triethanolamine salicylate. These ingredients play key roles in providing UV protection and stability to sunscreen formulations, but their use is restricted in some regions, such as India, due to regulatory differences [103].

In the ASEAN region, sunscreen products are regulated under the ASEAN Cosmetic Directive, with UV filters listed in Annex VII of the 2009 ASEAN Cosmetics Document. This document outlines the approved UV filters and the conditions for their use in cosmetic products. Importantly, the document also mandates those warnings. For example, “Avoid prolonged sun exposure, even when using a sunscreen product,” must be clearly displayed on the product labels to emphasize the importance of not relying solely on sunscreens for sun protection [104].

It’s worth noting that some UV filters listed in the ASEAN guidelines are not included in India’s list of approved UV filters for sunscreens. This reflects the differences in regulatory frameworks across regions, where certain ingredients may be approved in one jurisdiction but not in another, leading to variations in the types of UV protection available in cosmetic products. In India, the Bureau of Indian Standards (BIS) has specified the UV filters that are allowed to be included in cosmetic products. The Directive does not establish a maximum SPF rating. Protecting the skin from their harmful effects. However, UV filters used in cosmetic products solely to protect the product itself from UV rays are not included in this list [105].

While nanotechnology offers significant benefits in sunscreen formulations, including improved UV protection and aesthetic appeal, it also raises regulatory and safety concerns—particularly for ingredients with particle sizes below 100 nanometers. In certain regulatory jurisdictions, such as the European Union, any cosmetic product containing nanomaterials must explicitly label these on the ingredient list with the term “(nano)” after the substance name. Moreover, under EU Regulation (EC) No. 1223/2009, ingredients under 100 nm are subject to additional safety assessments and must be notified to the Cosmetic Products Notification Portal (CPNP). The European Scientific Committee on Consumer Safety (SCCS) has emphasized potential concerns regarding skin penetration, cytotoxicity, and bioaccumulation of nanoparticles. For example, although titanium dioxide and zinc oxide are approved as UV filters, their nano-sized forms are only permitted if shown not to penetrate viable skin layers and if their photoreactivity is minimal. In contrast, regions like the United States, Canada, and India have yet to implement unified guidelines specifically addressing nanoscale sunscreens, though ongoing evaluations by the FDA, Health Canada, and CDSCO are underway. These evolving regulatory frameworks underscore the need for robust safety data, transparent labeling, and risk assessments to ensure consumer and environmental safety when using nanostructured ingredients in cosmetic products [106].

## 8. Challenges

There are several challenges faced by manufacturers and consumers in using sunscreen. From the manufacturer’s perspective, one major hurdle is testing sunscreen formulations for both efficacy and toxicity, as well as ensuring compliance with labelling requirements set by regulatory authorities. When testing on human subjects, a significant issue arises from the carcinogenic effects of UV exposure, presenting ethical concerns for obtaining valid study data [107]. Additionally, factors like the high cost of sunscreen products can negatively affect sales, and the time required to develop a product that meets regulatory standards is another challenge manufacturers must navigate [108]. On the consumer side, one of the key challenges is the lack of understanding regarding the proper application of sunscreens. Labels often indicate the required amount of sunscreen for optimal protection, but many consumers fail to apply an adequate amount, resulting in reduced effectiveness of the product [109]. This inconsistency in application leads to varied results among different individuals. Maintaining consumer trust is another concern, as improper use can undermine the sunscreen’s photo-protective benefits. Additionally, the lack of uniformity in regulatory standards across countries creates confusion for both manufacturers and consumers. Different testing protocols and labelling requirements can lead to discrepancies in product effectiveness [110]. In developing countries, limited resources for producing sunscreen formulations add to the challenges, as both manufacturers and consumers face higher costs, further complicating access to effective sun protection. Despite significant advancements in nanotechnology and its successful application in the cosmetic industry, there have been very few clinical trials involving nano-formulations in sunscreens. One clinical trial developed a bio-adhesive nanoparticle-based sunscreen, claiming that the formulation prevented UV-induced damage by using nanoparticles as a delivery system. The bio-adhesive properties of the nanoparticles were said to provide extended protection. Additionally, incorporating the UV filter into the nanoparticle (BNP) helped prevent filter degradation, improved photo-stability, thereby reducing the likelihood of epidermal toxicity and cellular harm (NCT02668536) [111].

## 9. Future Perspectives

The future of sunscreen development lies in the integration of smart nanotechnology, artificial intelligence, and sustainable practices to create multifunctional and environmentally responsible formulations. AI-assisted nanocarrier design, using machine learning and predictive modelling, is accelerating the optimization of sunscreen ingredients for enhanced efficacy and reduced toxicity. Innovative UV-responsive delivery systems, triggered by sunlight, are being explored to provide on-demand release of antioxidants and UV filters, improving protection and reducing overexposure. There is a growing focus on multifunctional sunscreens that offer anti-aging, blue-light defense, and DNA repair capabilities, often through encapsulated enzymes and natural antioxidants. Personalized sunscreen formulations based on genetic and skin microbiome profiles are also emerging. Sustainable nanomanufacturing using green synthesis, biopolymers, and plant-based excipients is gaining momentum to minimize environmental impact. Additionally, oral nano-supplements and wearable UV-monitoring technologies are being developed to complement topical protection and enable real-time exposure feedback. These innovations are expected to shape a new era of intelligent, safe, and sustainable sun protection.

## 10. Conclusions

Nanotechnology has revolutionized sunscreen formulations, offering improved UV protection, aesthetic appeal, and stability. The combination of nanoparticles enhanced sun protection while addressing consumer preferences for transparency and skin compatibility. However, challenges such as environmental impact, public perception, and regulatory complexities persist. This review highlights the need for sustainable innovation, emphasizing the importance of eco-friendly materials and robust safety studies. As research advances, nano-based sunscreens can provide comprehensive skin benefits while maintaining a balance between efficacy, safety, and environmental response.

## Figures and Tables

**Table 1:** Types of Ultraviolet (UV) Rays and Their Characteristics. The table categorizes UV rays into UVA, UVB, and UVC, highlighting their wavelength ranges, effects on the skin, and atmospheric penetration [11].

Type of Ultraviolet Rays	Wavelength (nm)	Effects and Characteristics
**Ultraviolet A (UVA)**	320–400	Leads to premature skin aging, wrinkles, and cellular harm. Contributes to genetic mutations and plays a role in certain skin cancers.
**Ultraviolet B (UVB)**	280–320	Possesses slightly greater energy than UVA. Directly harms DNA in skin cells, leading to sunburn and a heightened risk of skin malignancies.
**Ultraviolet C (UVC)**	200–280	Extremely potent and hazardous. Naturally filtered out through ozone layer by preventing it from surface of earth. Man-made sources include welding torches, mercury vapor lamps, and UV disinfection bulbs, commonly used for eliminating microbes and pathogens.

**Table 2:** Frequency of usage—natural cosmetic ingredients from terrestrial sources. This table highlights the most commonly used species in cosmetics, along with their respective International Nomenclature of Cosmetic Ingredients (INCI) names and descriptions of their properties and benefits.

Class	Usage (%)	INCI	Explanation	References
*Helianthus annuus*	34 (7.7%)	*Helianthus annuus* Seed Oil	Extracted from sunflower seeds, Helianthus Annuus Seed Oil is derived from *Helianthus annuus* L., a member of the Compositae family.	[36]
Glycine max (oil)	30 (6.8%)	*Glycine soja* oil.	Obtained through extraction or pressing, Glycine Soja Oil is derived from the soybean (*Glycine soja*, Leguminosae) and contains triglycerides of oleic, linoleic, and saturated fatty acids.	[37]
Glycine max (extract)	3 (0.7%)	*Glycine Soja* Seed Extract	A natural extract obtained from soybean (*Glycine soja*, Leguminosae).	[38]
*Vitellaria paradoxa*	16 (3.6%)	*Butyrospermum parkii* butter	Extract of the seedcake of the Shea Tree (*Butyrospermum parkii*, Sapotaceae).	[39]
*Vitellaria paradoxa*	8 (1.8%)	*Butyrospermum Parkii* Butter Extract	Derived from the fruit of the Shea Tree (*Butyrospermum parkii*, Sapotaceae).	[40]
*Vitellaria paradoxa*	2 (0.5%)	*Butyrospermum Parkii* Butter Seedcake Extract	Extract sourced from the Shea Tree (*Butyrospermum parkii*, Sapotaceae).	[41]
*Persea gratissima*	11 (2.5%)	*Persea Gratissima* Fruit Extract	Extracted from the fruit of the avocado (*Persea gratissima*, Lauraceae).	[42]
*Persea gratissima*	11 (2.5%)	*Persea Gratissima* Oil	Fixed oil obtained by pressing the dehydrated sliced flesh of the avocado pear (*Persea gratissima*, Lauraceae), consisting mainly of fatty acid glycerides.	[43]
*Glycyrrhiza inflata*	21 (4.7%)	*Glycyrrhiza Inflata* Root Extract	Derived from the roots of *Glycyrrhiza inflata* (Leguminosae).	[43]
*Tanacetum parthenium*	7 (1.6%)	*Chrysanthemum parthenium* extract	Extracted from the feverfew herb (*Chrysanthemum parthenium*, Asteraceae).	[44]
*Tanacetum parthenium*	9 (2.0%)	*Chrysanthemum parthenium* Flower Extract	Sourced from the flowers of feverfew (*Chrysanthemum parthenium*, Asteraceae).	[45]
*Scutellaria baicalensis*	8 (1.8%)	*Scutellaria Baicalensis* Extract	Extract derived from the whole plant *Scutellaria baicalensis* (Lamiaceae).	[46]
*Scutellaria baicalensis*	8 (1.8%)	*Scutellaria Baicalensis* Root Extract	Obtained from the roots of *Scutellaria baicalensis* (Lamiaceae).	[47]
*Aloe barbadensis*	2 (0.5%)	*Aloe Barbadensis* Leaf Extract	Derived from the leaves of aloe (*Aloe barbadensis*, Liliaceae).	[48]
*Aloe barbadensis*	5 (1.1%)	*Aloe Barbadensis* Leaf Juice	Juice extracted from the leaves of *Aloe barbadensis* (Liliaceae).	[49]
*Aloe barbadensis*	6 (1.4%)	*Aloe Barbadensis* Leaf Juice Powder	Powdered form obtained from the dried juice of *Aloe barbadensis* (Liliaceae).	[49]

**Table 3:** Different nanosystems offer varying levels of UV protection, skin penetration, biocompatibility, and environmental safety. This provides a concise comparison to help guide the selection of suitable carriers in sunscreen formulations.

Nanosystem	UV Protection	Skin Penetration	Environmental Risk	Biocompatibility
Liposomes	Moderate	High	Low	High
SLNs/NLCs	High	Moderate	Moderate	High
Dendrimers	Very High	Very High	Unknown	Variable
Nanoemulsions	Moderate	High	Moderate	High

**Table 4:** Recent Patents on Sunscreen Innovations. This table presents newly patented sunscreen formulations, including their active ingredients, UV protection mechanisms, and potential benefits for enhanced skin protection.

Patent No.	Date of Patent	Work	Reference
202441088941	22/11/2024	Formulation design of herbal gel-based sunscreen containing silymarin from milk thistle and other ingredients to enhance UV protection and other applications.	[91]
202441081252	25/10/2024	A method of evaluating herbal sunscreen cream	[91]
202411071590	22/09/2024	Herbal-based sunscreen formulation and a method of preparation thereof	[91]
202441056209	24/07/2024	Herbal sunscreen formulation	[91]
202441048923	26/06/2024	Eco–friendly and biocomposite ZnO nanoparticle production for sunscreen and therapeutic formulations	[91]
202411048040	21/06/2024	Herbal sunscreen formulation comprising cucumber, tomato, and aloe vera extracts	[91]
202412033703	28/04/2024	Nano-lignin-based sunscreen composition, and a method of preparation thereof	[91]
202411019446	16/03/2024	Nano-sunscreen cream with ursolic acid-loaded solid lipid nanoparticles and method thereof	[91]
202417013840	26/02/2024	Sunscreen or daily care composition comprising bis-ethylhexyloxyphenol, methoxyphenyl triazine, and inorganic UV filters	[91]
202421011132	22/03/2024	Formulation and evaluation of sunscreen stick	[91]
202441008561	23/02/2024	Quercetin-loaded nanoliposomal UVA and UVB sunscreen cream formulation method	[91]
202341090278	31/12/2023	A green synthesis of sunscreen lotion and its product thereof	[91]
202341086711	19/12/2023	Sunscreen jacket	[91]
US2024385034A1	2024-11-21	Protective band to prevent skin damage to drivers	[92]
WO2024233498A1	2024-11-14	Water-in-oil emulsion metal oxide-based sunscreen formulations	[88]
KR102725430B1	2024-11-06	Cosmetic composition for sunscreen containing lignin modified with a silane-based surface modifier	[88]
